# Production and Selection of Antibody–Antigen Pairs for the Development of Immunoenzyme Assay and Lateral Flow Immunoassay Methods for Carbofuran and Its Analogues

**DOI:** 10.3390/bios12080560

**Published:** 2022-07-24

**Authors:** Yuxiang Wu, Qi Fan, Yinuo Chen, Xia Sun, Guoqing Shi

**Affiliations:** 1 School of Agricultural Engineering and Food Science, Shandong University of Technology, Zibo 255049, China; lvdukeji@126.com; 2 Shandong Lvdu Biotechnology Co., Ltd., Binzhou 256600, China; 3 School of Chemistry and Biological Engineering, University of Science and Technology Beijing, Beijing 100083, China; qi.fan17@outlook.com (Q.F.); 18612203588@163.com (Y.C.)

**Keywords:** carbofuran, benfuracarb, carbosulfan, immunoassay, monoclonal antibody

## Abstract

To produce a sensitive monoclonal antibody (mAb) for the simultaneous detection of carbofuran, benfuracarb, carbosulfan and 3-hydroxy-carbofuran, 2,3-dihydro-2,2-dimethyl-7-benzofuranmethanamine (DDB) was conjugated to bovine serum albumin (BSA) to prepare the immunogen DDB-BSA and mice were immunized. Coating antigens were prepared by conjugating DDB and 5-methoxy-2,3-dihydrobenzofuran-3-acetic acid (MDA) to BSA and ovalbumin (OVA), respectively. Furthermore, the effect of different antibody–antigen pairs on the sensitivity of ELISA and LFIA methods for the detection of carbofuran was investigated. After the immunization, a high-affinity mAb 13C8 was obtained. The ability of the coating antigen to compete with carbofuran for binding antibodies was found to be significantly different between ELISA and LFIA methods. With the antibody–antigen pair 13C8-MDA-OVA, the IC_50_ values of the ELISA and QD-LFIA methods for carbofuran were 0.18 ng/mL and 0.67 ng/mL, respectively. The cross-reactivity (CR) values of the two methods for benfuracarb, carbosulfan and 3-hydroxy-carbofuran ranged from 72.0% to 83.7%, while, for other carbamate pesticides, the CR values were less than 1%. The spiked recoveries of carbofuran in vegetables by the QD-LFIA method were 83–111%, with a coefficient of variation below 10%, and the test results of the actual samples were consistent with the HPLC-MS method. Overall, this study provides key materials for the development of immunoassays for carbofuran and its analogues, and the antibody–antigen pair selection strategy established in this study provides useful insights for the development of sensitive immunoassays for other compounds.

## 1. Introduction

**Figure 1:**
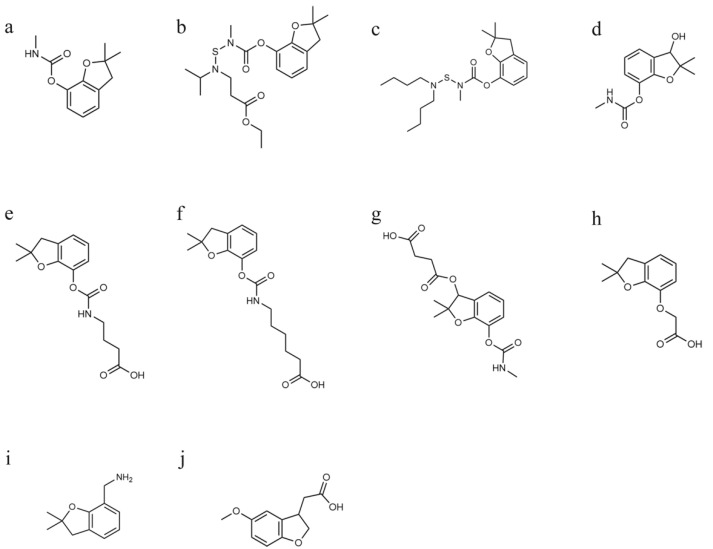
Chemical structures of carbofuran and its analogues. (**a**) Carbofuran. (**b**) Benfuracarb. (**c**) Carbosulfan. (**d**) 3-Hydroxy-Carbofuran. (**e**) BFNB. (**f**) BFNH. (**g**) 3-Succinyl-Carbofuran. (**h**) BFOA. (**i**) DDB. (**j**) MDA.

Carbofuran (Figure 1a), benfuracarb (Figure 1b) and carbosulfan (Figure 1c) are carbamate pesticides that were once widely used around the world [1]. Benfuracarb and carbosulfan have relatively lower toxicity than carbofuran; however, they can be metabolized to carbofuran and 3-hydroxy-carbofuran in the environment [2,3]. Carbofuran has an unacceptable risk for soils [4] and living organisms, including mammals [5,6], aquatic organisms [7,8] and birds [9]; therefore, many countries and regions, including China, the European Union and the United States, have withdrawn the registration of these three pesticides for fruits and vegetables in recent years [10,11,12,13,14], and a maximum residue limit (MRL) for these three pesticides has been set by different governments and organizations. For example, in the case of green vegetables, the MRLs for carbofuran are 0.02 mg/kg in China [15] and 0.1 mg/kg in the USA [16]. However, because these compounds have a better control effect on certain pests, there are sometimes irregularities in their use, resulting in the detection and exceedance of carbofuran residues in vegetables and fruits [17,18], water bodies [19] and soils [20]. To enhance the monitoring of the illegal use of carbofuran and its derivative pesticides, sensitive, rapid detection methods need to be established.

Rapid detection methods for carbofuran and its derivative pesticides are usually specific antibody-based immunoassays against carbofuran, including immunochromatographic assays [21,22,23,24], enzyme-linked immunosorbent assays (ELISAs) [21,22,23,25,26,27,28,29] and immunosensors [30,31,32,33,34], which are rapid, sensitive and suitable for on-site application. For example, Zhang et al. [24] reported a double-label time-resolved fluorescent strip to detect carbofuran with a detection limit of 0.76 ng/mL. Zhao et al. [21] used direct competitive ELISA to detect carbofuran with an IC_50_ of 1.3 ng/mL. Sun et al. [34] prepared a label-free electrochemical immunosensor based on a sol–gel-entrapped antibody for the analysis of carbofuran with a detection limit of 0.33 ng/mL.

For the production of carbofuran antibodies, compounds with the characteristic structure of carbofuran and with reactive groups have been used as haptens. Abad et al. [26] produced a monoclonal antibody (mAb) with the immunogen 4-[[(2,3-dihydro-2,2-dimethyl-7-benzofuranyloxy)carbonyl]amino]butanoic acid (BFNB)-BSA and developed an ELISA method to detect carbofuran with the heterologous coating antigen 6-[[(2,3-dihydro-2,2-dimethyl-7-benzofuranyloxy)carbonyl]amino]hexanoic acid (BFNH)-OVA; the IC_50_ for carbofuran was 0.66 ng/mL. Yao et al. [23] produced a mAb with the immunogen BFNB-keyhole limpet hemocyanin (KLH) and developed an ELISA method to detect carbofuran with the homologous coating antigen BFNB-ovalbumin(OVA); the IC_50_ for carbofuran was 0.3 ng/mL. Lan et al. [28] prepared a mAb with the immunogen 3-succinyl-carbofuran-KLH and established an ELISA method to detect carbofuran with the homologous coating antigen 3-succinyl-carbofuran-OVA; the IC_50_ for carbofuran was 0.76 ng/mL. Chen et al. [22] produced a mAb with the immunogen BFNH-lactoferrin and reported an ELISA method to detect carbofuran with the heterologous coating antigen 2-((2,2-dimethyl-2,3-dihydro-1-benzofuran-7-yl)oxy)acetic acid (BFOA)-BSA; the IC_50_ for carbofuran was 1.4 ng/mL. The chemical structures of these haptens are shown in Figure 1e–h. The production of high-affinity antibodies and the screening of antibody–antigen pairs are essential to the development of sensitive immunoassays.

Through recognition of a group of compounds with similar chemical structures, broad-specificity mAbs can detect multiple targets simultaneously and conveniently. Zou et al. [35] reported a broad-specificity mAb with the immunogen BFNB-BSA that can bind to carbofuran, isoprocarb, propoxur, carbaryl and carbosulfan. The IC_50_ values of the ELISA for carbofuran and carbosulfan were 8.97 ng/mL and 115.80 ng/mL, respectively. The mAb prepared by Lan et al. [28] can bind to carbofuran and 3-hydroxy-carbofuran, and the IC_50_ values of the ELISA for carbofuran and 3-hydroxy-carbofuran were 0.76 ng/mL and 0.69 ng/mL, respectively. However, to the best of our knowledge, no mAb for the simultaneous and sensitive detection of carbofuran, benfuracarb, carbosulfan and 3-hydroxy-carbofuran has been reported.

Due to the ban on the use of carbofuran, benfuracarb and carbosulfan in crops, such as fruits and vegetables, the development of a highly sensitive monoclonal antibody for the simultaneous detection of carbofuran, benfuracarb, carbosulfan and their metabolite 3-hydroxy-carbofuran is valuable for establishing a rapid detection method for the illegal use of the three pesticides mentioned above. In this study, 2,3-dihydro-2,2-dimethyl-7-benzofuranmethanamine (DDB, Figure 1i) was selected as the hapten because it has a 2,2-dimethyl-2,3-dihydro-1-benzofuran group, which is common in the three pesticides and their metabolites. To the best of our knowledge, DDB and its derivatives have not been used as haptens in the production of antibodies or immunoassays for carbofuran and its analogues. Then, the conjugate DDB-BSA was prepared as an immunogen to produce a monoclonal antibody. With the heterologous coating antigens 5-methoxy-2,3-dihydrobenzofuran-3-acetic acid (MDA)-BSA and MDA-OVA (Figure 1j) and the homologous coating antigens DDB-BSA and DDB-OVA, the effect of different antibody–antigen pairs on the detection of the targets by ELISA and LFIA was compared.

## 2. Materials and Methods

### 2.1. Materials and Instruments

Carbofuran, benfuracarb, carbosulfan and 3-hydroxy-carbofuran were purchased from TMRM Co., Ltd. (Beijing, China), while 1,1’-Carbonyldiimidazole (CDI) and OVA were purchased from Solarbio Science & Technology Co., Ltd. (Beijing, China). BSA and goat anti-mouse IgG conjugated with horseradish peroxidase were purchased from Sigma–Aldrich (St. Louis, MO, USA). N-hydroxysuccinimide (NHS), N-3-dimethylaminopropyl-N-ethylcarbodiimide hydrochloride (EDC) and dicyclohexylcarbodiimide (DCC) were purchased from Shanghai Yuanye Biotechnology Co., Ltd. (Shanghai, China). DDB was purchased from Thermo Fisher Scientific (Waltham, MA, USA). MDA was purchased from Accela ChemBio Co., Ltd. (Shanghai, China). The SPA column was purchased from Changzhou Smart-Lifesciences Biotechnology Co., Ltd. (Changzhou, China). Quantum dots (QDs, mean diameter: 300 nm, maximum emission wavelength: 525 nm) were obtained from Landu Biotechnology Co., Ltd. (Binzhou, China). Colloidal gold nanoparticles were prepared in our lab using the sodium citrate method, and the mean diameter was 20 nm. BALB/c mice were obtained from the National Laboratory Animal Centre, Qingdao Agricultural University, Qingdao, China. The XYZ3210 dispense system was purchased from Bio-Dot (Irvine, CA, USA). A Multiskan FC microplate photometer was purchased from Thermo Fisher Scientific (Shanghai, China). The QD reader was provided by Shandong Lvdu Biotechnology Co., Ltd. (Shandong, China). The high-performance liquid chromatography–tandem mass spectrometry device (HPLC-MS) was purchased from Agilent Technologies, Inc. (Santa Clara, CA, USA).

### 2.2. Syntheses of Immunogens and Coating Antigens

**Figure 2:**
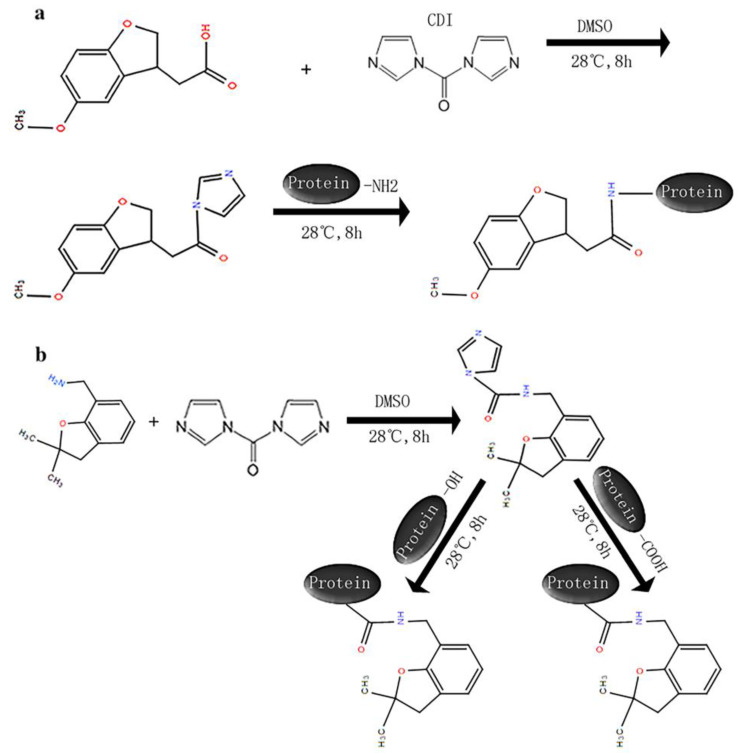
The synthetic routes for the immunogen and coating antigen. (**a**) Conjugation progress of hapten MDA to carrier protein (**b**) Conjugation progress of hapten DDB to carrier protein.

The synthetic routes for the immunogen and coating antigen are shown in Figure 2. The details are as follows: CDI (100 mg) and MDA (50 mg) were dissolved in 5 mL of dimethyl sulfoxide (DMSO) and shaken at 28 °C and 180 rpm for 8 h. The reaction products were added dropwise to 50 mL of BSA solution (10 mg/mL, in 0.05 M borate buffer) and shaken at 28 °C and 180 rpm for 8 h. After the reaction, the solution was dialyzed with 0.01 M phosphate-buffered saline (PBS) (pH 7.4) for more than 18 h to obtain the immunogen MDA-BSA. MDA-OVA, DDB-BSA and DDB-OVA were synthesized with a similar method.

### 2.3. Production of mAb

The mAb was prepared by the method previously described in our work [36], which was modified from the method described by Woychik et al. [37]. In brief, DDB-BSA was emulsified with an equal volume of propolis adjuvant instead of traditional Freund’s adjuvant. Then, male (8-week-old) BALB/c mice were injected intramuscularly with 50 μg DDB-BSA. The mice were boosted and immunized with the same amount of immunogen at the same site every week. Three to five days after the fifth immunization, the antibody titer and affinity were detected by indirect competitive ELISA (icELISA) with DDB-OVA as the coating antigen.

Spleen cells of the mouse that showed the best titers were fused with Sp2/0 myeloma cells. The desired antibodies with the highest affinity were screened by icELISA with carbofuran as a competitive compound, and hybridoma cells were cloned by the limited dilution method. Then, the selected hybridoma cells were injected into mice for ascites production. Finally, the mAbs were purified by (NH_4_)_2_SO_4_ with saturation of 50% and further purified by an SPA column.

### 2.4. Development of ELISA Method

Microplates were coated with coating antigens. Different concentrations of the standard (50 µL) and the mAb (50 µL) were added to each well, and the plates were incubated for 20 min at 37 °C. The plates were washed with PBS (0.01 M, containing 0.05% (*v*/*v*) Triton X-100), 100 μL of goat anti-mouse IgG-HRP conjugate solution was added to each well, and the plates were incubated for 20 min at 37 °C. After the plates were washed with PBS (0.01 M, containing 0.05% Triton X-100, *v*/*v*), 100 µL of TMB single-component substrate solution was added to each well, followed by incubation at 37 °C for 10 min. Finally, the reaction was terminated, and the absorbance at 450 nm was measured by a microplate reader.

### 2.5. Development of Colloidal Gold-Based LFIA Method

Ten milliliters of colloidal gold solution was adjusted to pH 8.0 with K_2_CO_3_ (0.1 M) at room temperature, and then 60 μg of the antibody was added. After mixing for 30 min at room temperature, 200 μL of 20% (*w*/*v*) BSA was added to block the free colloidal gold for 20 min at room temperature. Then, the mixture was centrifuged at 15,777× *g* for 30 min at 4 °C. Finally, the precipitate was resuspended in 2000 μL of PBS (0.005 M at pH 8.2, containing 10% BSA, 2% glucose, 0.5% alanine, 5% PEG2000 and 0.1% NaN_3_, *w*/*v*) and stored at 4 °C.

An immunochromatographic test strip consisted of a sample pad, a conjugate pad, a nitrocellulose (NC) membrane and an absorbent pad. The mAb–colloidal gold conjugates (700 μL) were sprayed onto the conjugate pad (6 mm × 300 mm) and then dried under vacuum for 20 h at room temperature. DDB-OVA (50 μg/mL) or MDA-OVA (30 μg/mL) and goat anti-mouse IgG antibodies were dispensed on the NC membrane at a rate of 1 μL/cm as the test line (T line) and control line (C line), respectively. The NC membrane was then dried at 37 °C for 3 h. The sample pads, conjugate pads, NC membranes and absorbent pads were attached to the PVC plastic support, and then the assembled plate was cut to obtain 4 mm test strips and placed in a rigid plastic cassette.

### 2.6. Development of Quantum Dot-Based LFIA Method

One milliliter of QDs was added to 9 mL of MES (0.054 M at pH 6.0) and centrifuged at 10,956× *g* for 20 min. Then, the precipitate was resuspended in 10 mL of MES (0.05 M at pH 6.0). After dispersion by ultrasonication for 2 min, EDC (20 mg) and NHS (20 mg) were added, and the reaction mixture was incubated for 30 min, followed by centrifugation at 10,956× *g* for 20 min. The precipitate was resuspended in 10 mL of MES (0.05 M at pH 6.0). Then, 1 mg of the mAb dissolved in 10 mL MES (0.027 M at pH 8.0) was added to the QD mixture and shaken at 25 °C and 180 rpm for 90 min. After the reaction, 5 mL of MES (0.054 M at pH 6.0 containing 1.6% BSA, *w*/*v*) was added, and the mixture was shaken at 25 °C and 180 rpm for 40 min, followed by centrifugation at 10,956× *g* for 20 min. The precipitate was resuspended in 10 mL of PB (0.01 M at pH 6.0, containing 5% casein, 1% glucose, 1.5% alanine and 0.05% NaN_3_, *w*/*v*) and dispersed by ultrasonication for 2 min.

The conjugate pad (6 mm × 300 mm) was pretreated with PBS (containing 0.5% Triton X-100, 0.5% alanine and 0.05% NaN_3_, *w*/*v*). Then, 1 mL of the mAb–QD conjugate was sprayed onto the pretreated conjugate pad at a flow rate of 10 μL/cm, and the pad was dried at 37 °C for 2 h. Other applied protocols were similar to those mentioned above.

### 2.7. Standard Curves and IC_50_ for ELISA and QD-LFIA Methods

Taking the logarithm of the carbofuran concentration as the abscissa and (B/B_0_)% as the ordinate, the standard curves for ELISA and QD-LFIA were obtained based on the Morgan–Mercer–Flodin (MMF) model, which were fitted by CurveExpert 1.4.0 software (https://www.curveexpert.net, accessed on 15 July 2022). For the ELISA method, B and B_0_ are the absorbance of the sample well and blank well, respectively. For the QD-LFIA method, B and B_0_ are the T/C (fluorescence intensity of the test line/fluorescence intensity of the control line) of the sample and blank control, respectively.

The inhibition ratio (I%) was calculated as (B_0_-B)/B_0_ × 100%. IC_50_ is the concentration of the carbofuran at an I% of 50%, and the limit of detection (LOD) was determined as the concentration showing 10% of inhibition.

### 2.8. Cross-Reactivity

The cross-reactivities (CRs) of benfuracarb, carbosulfan, 3-hydroxy-carbofuran, metolcarb, carbaryl, isoprocarb and aldicarb with carbofuran were assessed by ELISA and QD-LFIA. CR was calculated as CR (%)  =  [IC_50_ (carbofuran)/IC_50_ (analogues)] × 100%.

### 2.9. Analysis of Vegetable Samples by QD-LFIA Method

The accuracy and precision of the QD-LFIA method were assessed by the spike and recovery tests. Celery samples spiked with carbofuran with concentrations of 10, 20 and 50 ng/g were detected by the QD-LFIA method. The celery sample was homogenized, and then 5 ± 0.05 g was weighed and added in a 50 mL centrifuge tube. Then, 10 mL of methanol–water (80:20, *v*/*v*) was added and the mixture was vigorously mixed for 1 min to extract carbofuran, followed by centrifugation at 7012× *g* for 5 min. Finally, 100 μL of supernatant was diluted with 400 μL of sample diluent (0.9% NaCl solution, *w*/*v*).

### 2.10. Comparison with HPLC-MS Methods

Ten celery samples were detected by the QD-LFIA and HPLC-MS methods. The HPLC-MS procedures were performed according to the standard method [38]. Briefly, 5 g samples were extracted with 20 mL of acetonitrile and oscillated for 30 min, followed by centrifugation at 2739× *g* for 5 min. Extraction was repeated twice and supernatants were combined. Then, 50 mg of primary secondary amine (PSA) was added to 2 mL of supernatant and vortexed for 30 s, followed by centrifugation at 2739× *g* for 3 min. Subsequently, 1 mL of supernatant was blown with nitrogen at 40 °C until nearly dry. The residue was redissolved in 0.025% (*w*/*v*) ammonia solution (containing 30% acetonitrile, *v*/*v*) and vortexed. The extract was filtered through a 0.22 μm filter membrane for analysis. The separation was performed on a C_18_ column at 35 °C. The measurement was performed via electrospray ionization in positive mode. The mean results of the two methods were compared to verify the accuracy and reliability of the QD-LFIA method.

## 3. Results and Discussion

### 3.1. Production of mAb

After cell fusion, for the mice immunized with DDB-BSA, 980 positive cell strains were screened by ELISA with the coating antigen DDB-OVA at a carbofuran concentration of 50 ng/mL, and six cell lines that showed higher I% were selected and cloned by the limited dilution method. Then, the reactivity of the supernatant of the six cell lines to 50 ng/mL carbofuran, benfuracarb and carbosulfan was assessed by ELISA, and the results are shown in Table 1. The mAbs produced by all six cell lines showed reactivity to the three pesticides, proving that DDB is a suitable hapten for the production of antibodies recognizing the common 2,2-dimethyl-2,3-dihydro-1-benzofuran group in carbofuran, benfuracarb and carbosulfan. Because 13C8 had the highest reactivity with carbofuran, benfuracarb and carbosulfan among the six cell lines, it was injected into mice for ascites production, and the mAbs were purified by a protein A column for further experiments.

### 3.2. Screening of Antibody–Antigen Pairs

The microplate was coated with the coating antigens DDB-BSA (1 µg/mL), DDB-OVA (0.5 µg/mL), MDA-BSA (0.2 µg/mL) and MDA-OVA (0.1 µg/mL), and the concentration of mAb 13C8 was 0.03 µg/mL. Carbofuran standards (0, 0.05, 0.15, 0.45, 1.35 and 4.05 ng/mL) were detected to obtain a standard curve, and the IC_50_ was calculated. As shown in Table 2, the IC_50_ of the ELISA for carbofuran with different antibody–antigen pairs was between 0.17 and 0.22 ng/mL, indicating that different hapten (DDB and MDA) and carrier protein (BSA and OVA) pairs had similar sensitivities. In the icELISA used in this study, free antigen (carbofuran) in solution competed with the coating antigen on the microplate for antibody 13C8; therefore, the IC_50_ value was negatively correlated with the antibody-binding ability of free antigen competing with coating antigens. The results in Table 2 show that there was no significant difference in the antibody-binding ability of carbofuran competing with the four coating antigens in ELISA.

Then, to prepare QD-LFIA test strips, DDB-BSA (40 μg/mL), DDB-OVA (50 μg/mL), MDA-BSA (35 μg/mL) and MDA-OVA (30 μg/mL) were dispensed onto the NC membrane, and antibody 13C8 was conjugated to the QDs. Carbofuran standards (0, 0.05, 0.15, 0.45, 1.35 and 4.05 ng/mL) were detected to obtain a standard curve, and the IC_50_ was calculated. The results (Table 2) show that QD-LFIA test strips prepared with homologous coating antigens (DDB-BSA and DDB-OVA) had almost no reaction to carbofuran, while those prepared with heterologous coating antigens (MDA-BSA and MDA-OVA) detected carbofuran sensitively (IC_50_ values of 0.66–0.68 ng/mL). In LFIA, carbofuran in solution competed with the coating antigen on the NC membrane for antibody 13C8 conjugated to the QDs. The antibody-binding ability of carbofuran competing with coating antigens was affected by the immobilization of coating antigens and the conjugation of antibodies. Comparing the IC_50_ values of ELISA and LFIA, we can conclude that in LFIA, the antibody-binding ability of carbofuran competing with heterologous coating antigens decreased slightly, while that of carbofuran competing with homologous coating antigens decreased significantly.

**Figure 3:**
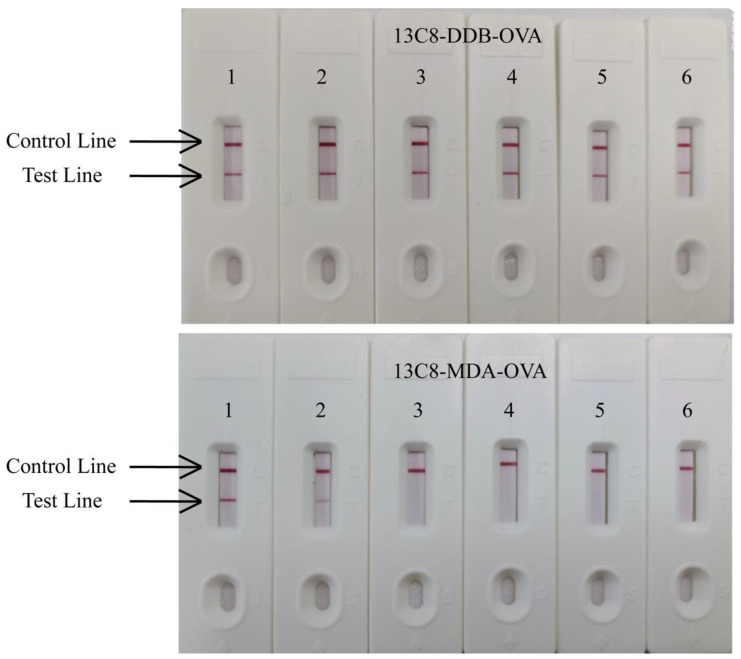
The sensitivity of colloidal gold-based lateral flow immunoassay for carbofuran with 13C8 and different coating antigens. 1  =  0 ng/mL, 2  =  1 ng/mL, 3  =  3 ng/mL, 4  =  5 ng/mL, 5  =  100 ng/mL, 6  =  1000 ng/mL.

We also dispensed DDB-OVA (50 μg/mL) and MDA-OVA (30 μg/mL) onto the NC membrane and labeled antibody 13C8 with colloidal gold to prepare colloidal gold LFIA test strips. The reaction of the test strip to different concentrations of carbofuran is shown in Figure 3. Similar to QD-LFIA, T lines of colloidal gold LFIA test strips prepared with the homologous coating antigen DDB-OVA had almost no change at 1000 ng/mL carbofuran compared with that at 0 ng/mL. However, the T lines of the test strips prepared with the heterologous coating antigen MDA-OVA became significantly lighter at 1 ng/mL carbofuran compared with that at 0 ng/mL, and a cut-off of 3 ng/mL was established. These experimental results further prove that antibody 13C8–antigen MDA-BSA pairs are suitable for LFIA.

ELISA is often used to screen highly sensitive mAbs; however, the sensitivity sometimes decreases significantly when the mAbs are used in LFIA. Our study shows that it is feasible to maintain the antibody-binding ability of free antigens by using heterologous coating antigens.

### 3.3. Detection of Carbofuran by ELISA

**Figure 4:**
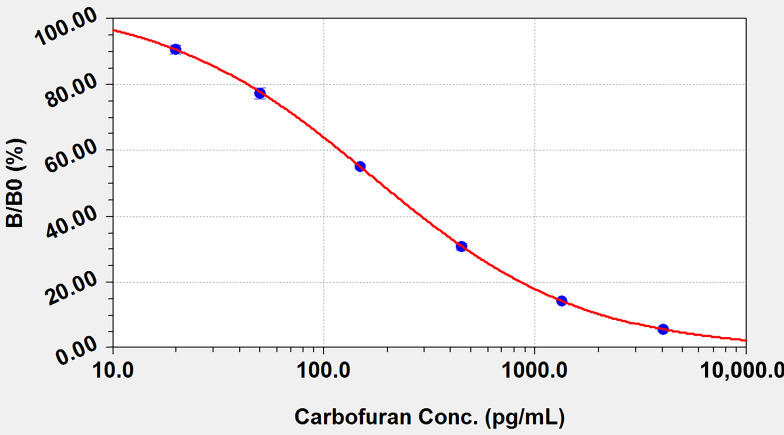
Standard curve for the determination of carbofuran by ELISA; the abscissa is the logarithm of the carbofuran concentration (pg/mL) and the ordinate is the inhibition rate.

The ELISA method was developed with the antibody 13C8–antigen MDA-BSA pair. Under the optimized coating antigen concentration (0.1 μg/mL), HRP-conjugated goat anti-mouse IgG concentration (1 μg/mL), antibody concentration (0.03 μg/mL) and working buffer parameters (5 mM PBS at pH 7.4), a standard curve was obtained with the concentration of carbofuran on the abscissa and the inhibition ratio (B/B_0_) on the ordinate. As shown in Figure 4, the standard curve equation is y = (a × b + c × x^d)/(b + x^d), in which a = 105.14, b = 83.63, c = −0.79 and d = 0.86; x is the concentration of carbofuran, and y is B/B_0_. The working range of the carbofuran concentration was found to be 0.02–2.09 ng/mL, with an IC_50_ of 0.18 ng/mL and a detection limit of 0.02 ng/mL. Compared with current methods, the IC_50_ and the LOD of our method with the screened antibody–antigen pairs were lower.

Standard curves for the determination of benfuracarb, carbosulfan, 3-hydroxy-carbofuran, metolcarb, carbaryl and aldicarb were obtained under the optimized ELISA conditions, and the IC_50_ and CR were calculated. As shown in Table 3, the cross-reactivities with benfuracarb, carbosulfan and 3-hydroxy-carbofuran were all above 70%, while those with metolcarb, carbaryl and aldicarb were all under 1%, proving that this method can be used to detect the total amount of carbofuran, its analogues and their main metabolite. Compared with previous literature, the ic-ELISA methods presented in this work had higher sensitivity and higher reaction consistency to carbofuran derivatives (Table 4).

### 3.4. Detection of Carbofuran by QD-LFIA

The QD-LFIA method was developed with the antibody 13C8–antigen MDA-BSA pair. The optimized coating antigen concentration on the T line was 30 μg/mL. A standard curve was developed with the inhibition ratio (B/B_0_) versus the logarithm of the concentration of carbofuran.

**Figure 5:**
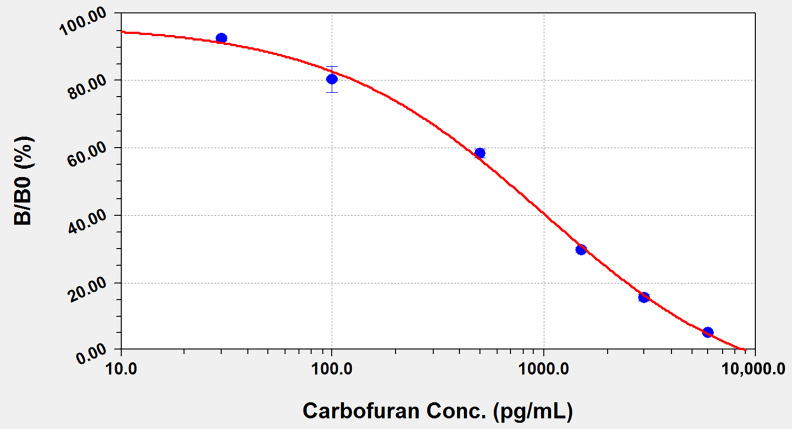
Standard curve for the determination of carbofuran by QD-LFIA; the abscissa is the logarithm of the carbofuran concentration (pg/mL) and the ordinate is the inhibition rate.

As shown in Figure 5, the standard curve equation is y = (a × b + c × x^d)/(b + x^d), in which a = 96.71, b = 349.36, c = −15.32 and d = 0.85; x is the concentration of carbofuran, and y is B/B_0_. The working range of the carbofuran concentration was found to be 0.04–4.21 ng/mL, with an IC_50_ of 0.67 ng/mL and a detection limit of 0.04 ng/mL.

Different concentrations of benfuracarb, carbosulfan, 3-hydroxy-carbofuran, metolcarb, carbaryl and aldicarb were detected to obtain standard curves, and the IC_50_ and CR were calculated. As shown in Table 5, the cross-reactivities with benfuracarb, carbosulfan and 3-hydroxy-carbofuran were all above 77%, while those with metolcarb, carbaryl and aldicarb were all under 1%, proving that this method can be used to detect the total amount of carbofuran, its analogues and their main metabolite.

### 3.5. Recovery of Carbofuran in Vegetable Samples by QD-LFIA Method

To verify the accuracy and precision of the QD-LFIA method, we performed spike and recovery tests at three carbofuran concentrations on celery samples with three repetitions. As shown in Table 6, the average recovery was 83–111%, with coefficient of variance (CV) values less than 10%, indicating that this method has good accuracy and precision.

### 3.6. Comparison with HPLC-MS Methods

To further verify the accuracy and reliability of the QD-LFIA method, 10 celery samples were detected by the QD-LFIA and HPLC-MS methods with two repetitions. The result shows that carbofuran was not detected (below the LOD of 2.5 ng/g) in all samples by the HPLC-MS method. For the QD-LFIA method, carbofuran and its analogues were detected in six samples, with residue levels ranging from 0.85 to 2.38 ng/g. The results of the two methods are essentially in good agreement, indicating that the developed QD-LFIA method can be used to detect carbofuran in vegetables.

## 4. Conclusions

In this study, we immunized mice with an immunogen that contained the 2,2-dimethyl-2,3-dihydro-1-benzofuran group, which is common in carbofuran, benfuracarb and carbosulfan, and developed a monoclonal antibody for the simultaneous detection of carbofuran and its analogues that is more sensitive than current methods. This study also found that, compared with homologous coating antigens, antibody–heterologous antigen pairs have similar sensitivity in ELISA and significantly higher sensitivity in LFIA. Under the tentatively optimized conditions, the IC_50_ values of the ELISA and QD-LFIA methods developed with the antibody 13C8–antigen MDA-BSA pair for carbofuran were 0.18 ng/mL and 0.67 ng/mL, respectively, and the cross-reactivity with analogues of carbofuran and its metabolite was above 72%. The spiked recoveries of carbofuran in vegetables by the QD-LFIA method were 83–111% with CV% below 10%, and the detection results in actual samples were consistent with the HPLC-MS method. Therefore, these methods can be applied to the rapid on-site detection of carbofuran and its analogues. Furthermore, the use of antibody–heterologous antigen pairs reported in this study offers important insights for improving the sensitivity of competitive LFIA.

## Figures and Tables

**Table 1:** Reactivity of mAbs produced by different cell lines to carbofuran, benfuracarb and carbosulfan.

Cell Lines	Carbofuran	Benfuracarb	Carbosulfan
13C8	95%	91%	90%
6D6	86%	53%	61%
12D6	84%	55%	46%
18G3	86%	76%	71%
12H5	78%	70%	67%
7F9	76%	68%	65%

**Table 2:** The effect of different antibody–antigen pairs on the IC_50_ of carbofuran by ELISA and LFIA.

Antibody	Antigen	IC_50_ (ELISA)ng/mL	IC_50_ (QD-LFIA) ng/mL
13C8	DDB-OVA	0.22 ± 0.02	>1000
	MDA-OVA	0.17 ± 0.01	0.66 ± 0.03
	DDB-BSA	0.19 ± 0.01	>1000
	MDA-BSA	0.20 ± 0.02	0.68 ± 0.03

**Table 3:** Cross-reactivity of ELISA between carbofuran and carbofuran analogues.

Chemicals	IC_50_	CR (%)
carbofuran	0.18 ± 0.03	100.0
benfuracarb	0.23 ± 0.03	78.3
carbosulfan	0.24 ± 0.04	75.0
3-hydroxy-carbofuran	0.25 ± 0.05	72.0
metolcarb	>20	<1%
carbaryl	>20	<1%
isoprocarb	>20	<1%
aldicarb	>20	<1%

**Table 4:** Performance comparison of ic-ELISA.

Works	Chemicals	IC_50_	CR(%)	Reference
1	carbofuran	0.3	/	[23]
2	carbofuran	0.66	/	[26]
3	carbofuran	0.76 ± 0.07	100.0	[28]
	3-hydroxy-carbofuran	0.69 ± 0.08	110.1	
4	carbofuran	8.97	100.0	[35]
	isoprocarb	17.68	50.7	
	propoxur	34.75	25.8	
	carbosulfan	115.80	7.7	
	carbaryl	105.59	8.5	
5	carbofuran	0.18 ± 0.03	100.0	this work
	benfuracarb	0.23 ± 0.03	78.3	
	carbosulfan	0.24 ± 0.04	75.0	
	3-hydroxy-carbofuran	0.25 ± 0.05	72.0	

**Table 5:** Cross-reactivity of QD-LFIA between carbofuran and carbofuran analogues.

Chemicals	IC_50_	CR (%)
carbofuran	0.67 ± 0.08	100.0
benfuracarb	0.81 ± 0.07	83.7
carbosulfan	0.84 ± 0.09	79.8
3-hydroxy-carbofuran	0.87 ± 0.06	77.0
metolcarb	>100	<1%
carbaryl	>100	<1%
isoprocarb	>100	<1%
aldicarb	>100	<1%

**Table 6:** Recoveries of carbofuran by the developed QD-LFIA.

Spiked Concentration (ng/g)	Measured Concentration (ng/g)	Recovery (%)	CV (%)
10	8.3 ± 0.6	83	6.9
20	20.4 ± 1.3	102	6.5
50	55.7 ± 4.9	111	8.7

## Data Availability

The data presented in this study are available on request from the corresponding author.
